# Modified triangulating stapling technique for esophagogastrostomy after esophagectomy for esophageal cancer

**DOI:** 10.1007/s00464-012-2586-8

**Published:** 2012-10-24

**Authors:** Masashi Takemura, Kayo Yoshida, Yushi Fujiwara

**Affiliations:** 1 Department of Upper Gastrointestinal Surgery, Hyogo College of Medicine, 1-1, Mucogawa-machi, Nishinomiya, Hyogo 663-8501 Japan; 2 Department of Gastrointestinal Surgery, Osaka City General Hospital, Osaka, Japan

**Keywords:** Benign stenosis, Esophageal cancer, Esophagogastric anastomosis, Triangulating stapling technique

## Abstract

**Background:**

Anastomosis performed during esophagectomy for esophageal cancer is usually involves hand-sewn or circular stapled methods. However, these techniques have been reported to be associated with a high frequency of anastomotic complications, including leakage and benign stenosis. Here a novel triangulating stapling technique for esophagogastrostomy after esophagectomy for esophageal cancer and its retrospective investigation are described.

**Methods:**

Forty-eight patients were underwent esophagectomy for esophageal cancer from January 2006 to December 2009 by the same surgeon using the triangulating stapling technique. The short-term outcomes were evaluated retrospectively. This end-to-end anastomosis used three linear staplers in an everted fashion.

**Results:**

Patients comprised 36 men and 12 women with a mean age of 59.4 years. Anastomotic leakage occurred in 4 patients (8.3 %), while anastomotic stenosis was observed in 6 (12.5 %). The average number of endoscopic pneumatic dilatations in patients with anastomotic stenosis was 2.4. The median (range) duration of hospital stay was 40.8 (19–154) days.

**Conclusions:**

Our modified triangulating stapling technique for esophagogastrostomy may be a feasible alternative, resulting in a lower frequency of postoperative anastomotic complications.

Transthoracic esophagectomy and reconstruction using gastric tube is considered the standard surgical treatment for patients with thoracic esophageal cancer. However, various surgical techniques have been reported for cervical esophagogastric anastomosis, which is conventionally performed by hand sewing [1–4]. The stapled anastomosis technique using a circular stapler has been recently introduced and is considered to be useful for shortening operation time and reducing anastomotic leakage [2, 5]. However, there were some problems in anastomosis using the circular stapler. Because the suturing device is inserted from the top of the gastric tube for end-to-side esophagogastric anastomosis, the circulation net of gastric wall was blocked by the stapler, causing suture failure. Furthermore, the frequency of benign anastomosis stenosis after esophagogastric anastomosis using the circular stapler is high, ranging from 5 to 40 % in the recent literature [2]. The benign anastomotic stenosis can cause swallowing dysfunction associated with a low quality of life for patients after esophagectomy [6, 7].

More recently, Furukawa et al. [8] reported the usefulness of triangular anastomosis using the linear stapler. In their anastomosis technique, one of the three sides is anastomosed in an inverted fashion, while the other two sides are anastomosed in an everted fashion. They showed that triangular-stapled anastomosis had a lower frequency of anastomotic failure and stenosis than that of hand-sewn or circular stapler anastomosis.

Since January 2006, we have performed a modified triangular stapled anastomosis using a linear stapler. In our technique, three sides are anastomosed in an everted fashion. In this report, we describe our surgical technique and evaluate the clinical results of this technique.

## Materials and methods

### Procedure

**Fig. 1:**
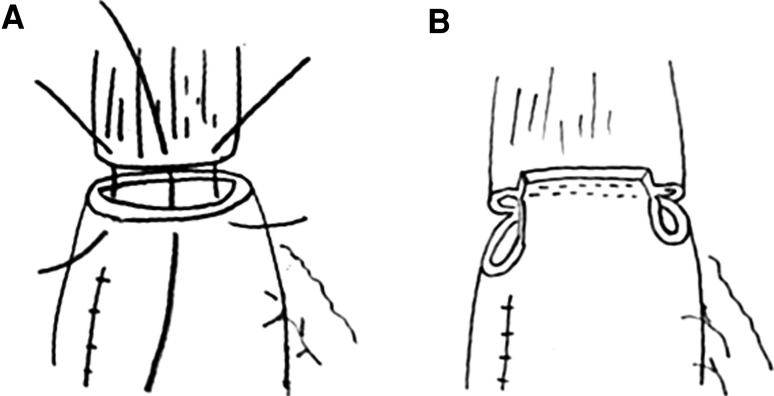
Modified triangulating stapling technique for esophagogastrostomy using linear staplers. The first linear stapler was applied after 3 or 4 stay sutures through the whole layer (**A**). These stay sutures were pulled up, and all stay sutures were then completely removed with a linear stapler (**B**)

**Fig. 2:**
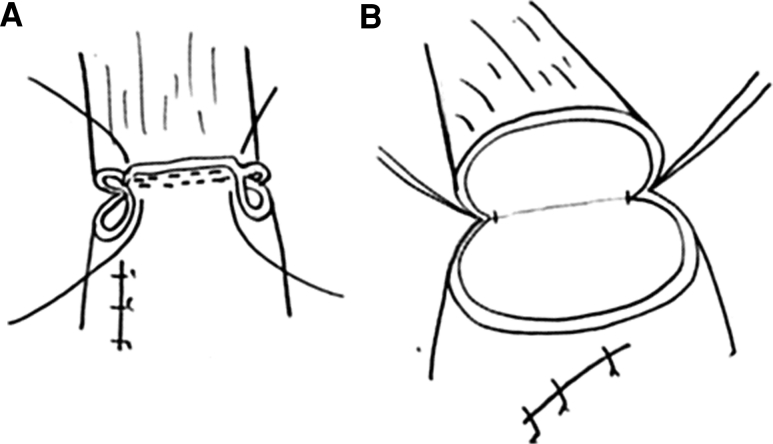
The stay sutures were applied at the both ends of the first anastomotic staple line (**A**). The anastomotic region was half-turned using the one thread of these sutures (**B**)

**Fig. 3:**
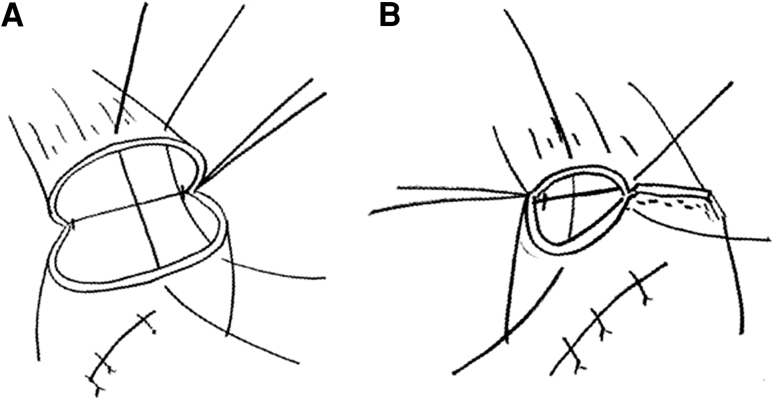
A stay suture was applied to the center of the posterior wall of the remnant esophagus and gastric tube in whole layer, and 1 or 2 more additional stay sutures were applied between the end of the first staple line at the anterior wall and the center of posterior wall (**A**). Half of the posterior wall was anastomosed evertly using a linear stapler. After the stay sutures were inserted to the end of second staple line, we pulled up the stay sutures and performed anastomosis with a third linear stapler (**B**)

**Fig. 4:**
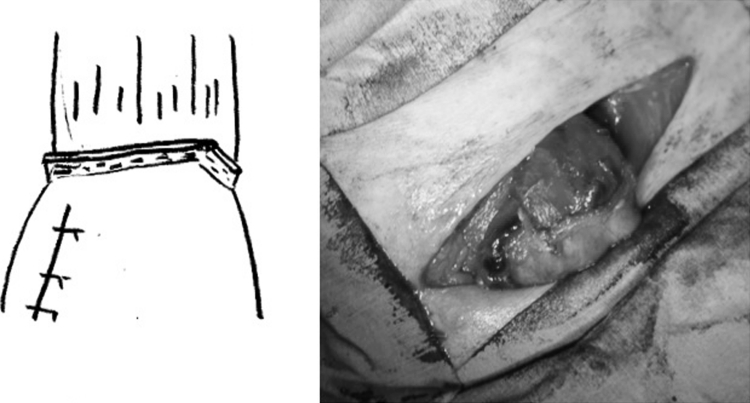
End-to-end anastomosis using modified triangulating stapling technique is completed. The gastric tube was pulled down from an abdominal cavity, and torsion was spontaneously relieved when the gastric tube returned to the intrathoracic cavity

We typically performed thoracic esophagectomy and mediastinal lymph node dissection in the left decubitus position via a thoracoscopic approach. After the thoracic procedure was completed, the position of the patient was changed to supine. A gastric tube was made using linear stapler via a laparoscopic approach. If the tumor invaded the upper thoracic esophagus, a bilateral cervical lymph node dissection was performed after the abdominal procedure. We used a narrow gastric tube that was sufficiently long to transverse the posterior mediastinal route to the cervix to serve as an esophageal substitute. Esophagogastric anastomosis was performed on the left side of cervix. A modified triangular stapled anastomosis was made using three linear staplers. The first instrumental anastomosis was applied to the anterior wall of the remnant esophagus and the superior end of the gastric tube in an everted fashion. The first linear stapler was applied after three or four stay sutures through the whole layer were added to secure the first anastomosis, and then these stay sutures were pulled up and all stay sutures were then completely removed with the linear stapler (Fig. 1A, B). We applied the stay sutures at the both ends of the first anastomotic staple line (Fig. 2A). The anastomotic region was half-turned using one thread of these sutures (Fig. 2B). Furthermore, a stay suture was applied to the center of the posterior wall of the remnant esophagus and gastric tube in the whole layer, and one or two more additional stay sutures were applied between the end of the first staple line at the anterior wall and the center of posterior wall (Fig. 3A). Half of the posterior wall was anastomosed evertly with a linear stapler while pulling up the stay sutures. The most important feature of this surgical technique was that the suture lines had to be securely intersected. After the stay sutures were inserted to the end of the second staple line, we pulled up the stay sutures and performed anastomosis with a third linear stapler (Fig. 3B). Then an anastomosis was completely formed evertly (Fig. 4). After confirming hemostasis, the gastric tube was pulled down from an abdominal cavity, and torsion was spontaneously relieved when the gastric tube returned to the intrathoracic cavity.

**Fig. 5:**
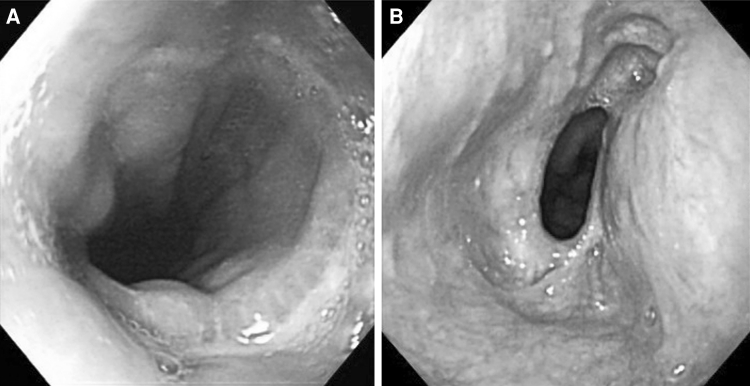
Postoperative endoscopic views of the anastomotic site. The lumen of the triangular anastomosis (**A**) was wider than that of the circular stapled anastomosis (**B**). All sides of the triangle were everted, and no mucosal defect was observed

The postoperative endoscopic views of the anastomotic site are shown in Fig. 5. The lumen of the triangular anastomosis (Fig. 5A) was wider than that of the circular stapled anastomosis (Fig. 5B). All sides of the triangle were everted, and no mucosal defect was evident.

### Patients

Medical records of 48 patients with thoracic esophageal cancer treated at our hospital from January 2006 to December 2009 were reviewed retrospectively. All patients were pathologically diagnosed with esophageal squamous cell carcinoma, and radical esophagectomies were performed. All of the operations were performed by a single surgeon (M.T.).

The clinical characteristics of the patients are shown in Table 1. Patients comprised 36 men and 12 women with a mean (range) age of 59.4 (42–77) years at the time of esophagectomies. The tumors were located in the upper thoracic esophagus in 3 patients, middle thoracic esophagus in 30, and lower thoracic esophagus in 15. The pathological stage classification was according to the Japanese Classification of Esophageal Cancer of the Japanese Society for Esophageal Diseases [9] and 4 patients had stage 0, 5 had stage I, 7 had stage II, 25 had stage III, and 7 had stage IV disease.

**Table 1:** Clinical characteristics of patients undergoing the modified triangulating stapling technique

Characteristic	Value
Age (years) (mean ± SD)	59.4 ± 8.6
Gender (*n*)	
Male	36
Female	12
Location of tumor (*n*)	
Upper third	3
Middle third	30
Lower third	15
Stage (*n*)	
0	4
I	5
II	7
III	25
IV	7
Thoracic procedure (*n*)	
Thoracoscope	48
Thoracotomy	0
Abdominal procedure (*n*)	
Laparoscope	44
Laparotomy	4
Operation time (min), mean ± SD (range)	440.2 ± 94.9 (262–784)
Operative blood loss (milliliters), mean ± SD (range)	486.1 ± 242.8 (120–1310)
Postoperative hospital stay (days), mean ± SD (range)	42.8 ± 28.1 (19–154)

The mean (range) operating time and estimated blood loss were 440 (262–784) minutes and 486 (120–1,310) milliliters, respectively. Postoperative barium swallowing was performed on the eighth postoperative day to evaluate the swallowing function and anastomotic leakage. If anastomotic leakage was diagnosed, endoscopic examination and chest computed tomography were immediately performed.

Frequencies of postoperative complications are shown in Table 2. Anastomotic leakage occurred in 4 patients (8.3 %). All patients with anastomotic leakage recovered quickly after draining from the cervical wound. Anastomotic stenosis was observed in 6 patients (12.5 %). The average number of endoscopic pneumatic dilatations in patients with anastomotic stenosis was 2.4. There was no hospital death in these patients. The median (range) length of hospital stay was 40.8 (19–154) days.

**Table 2:** Postoperative complications related to anastomosis of patients undergoing a modified triangulating stapling technique

Characteristic	*n* (%)
Anastomotic leakage	4 (8.3)
Laryngeal nerve palsy	7 (14.6)
Chylothorax	2 (4.2)
Pneumonia	2 (4.2)
Necrosis of gastric roll	1 (2.1)
Cardiovascular complication	1 (2.1)
Benign anastomosis stricture	6 (12.5)

## Discussion

The surgical treatment of esophageal cancer remains controversial [10]. For example, various reconstructive options are available after esophagectomy for esophageal cancer. These options depend on the reconstructed organ (stomach, colon, or small intestine), characteristics of the esophageal conduit (whole stomach or thin gastric roll, jejunum or ileum, or left or right colon), location of anastomosis (cervical or thoracic), anastomotic method (hand-sewn or stapled), route of reconstruction (antethoracic, retrosternal, or postmediastinum). Of these factors, the anastomotic technique is obviously one of many variables that can affect the operative morbidity or postoperative course. In fact, hospital deaths after esophagectomy are related to postoperative impediments, such as pulmonary complications and anastomotic leakage [11–13]. Therefore, much effort has been devoted to reducing the occurrence of anastomotic leakage.

The rate of anastomotic leakage of the anastomosis between the remnant cervical esophagus and esophageal substitute is higher than that of other type of gastrointestinal anastomosis. To date, many studies have compared the anastomotic complication of the hand-sewn or stapled anastomosis. Kim and Takabe [2] reviewed the major outcomes of the nonrandomized or randomized control trials of esophagogastric anastomosis after esophagectomy for esophageal cancer. In this review, several reports of nonrandomized studies described a decreased rate of anastomotic leakage with stapled anastomosis compared to hand-sewn anastomosis. However, none of the randomized control trials reported statistically significant differences in the rate of anastomotic leakage, which varied depending on the reconstructed organs, approach, or anastomotic technique.

Reports of the outcomes and usefulness of the triangular stapler technique for esophagogastric anastomosis are rare [3, 8]. Toh et al. [3] compared postoperative complications between the triangulating stapling technique and hand-sewn anastomosis. Their first stapling was performed on the posterior wall in an inverted fashion, and the other two sides of the anterior wall were stapled in an everted fashion. This report concluded that the triangulating stapling technique may reduce the frequency of anastomotic complications, including leakage or benign stenosis. On the other hand, Furukawa et al. [8] compared with the anastomotic-related complications among the hand-sewn anastomosis, circular stapling method, and triangulating anastomosis methods. The triangulating anastomosis technique in this study was the same as technique reported by Toh et al. [3]. In their report, the rate of anastomotic leakage of the triangulating anastomosis method was only 8.3 %, and the time required was significantly shorted compared to the other two techniques. In our triangulating technique, anastomosis of all sides were performed in an everted fashion. The rate of anastomotic leakage in our technique is 8.3 %, similar to previous reports.

Benign anastomotic stenosis causing dysphagia after esophagectomy is a burdensome complication that greatly impairs quality of life after surgery [6, 7]. Ischemia and anastomotic technique are the most important risk factors for benign stenosis [2, 14, 15]. Various incidences of anastomotic stenosis have been reported so far. Worrell et al. [16] analyzed the complications between stapled and hand-sewn anastomosis. In this report, the incidence of anastomotic stenosis was 38 % in the hand-sewn anastomosis compared to 26 % in the stapled anastomosis. Van Heijl et al. [6] studied large case series after esophagectomy to identify the independent risk factors for development of benign anastomotic stenosis. They reported that 41.7 % of patients developed a benign stenosis during the follow-up period. Cardiovascular disease and anastomotic leakage were independent predictors for the development of benign anastomotic stenosis. On the other hand, Furukawa et al. [8] reported that the anastomotic stenosis was observed in 8.3 % at triangulating stapled anastomosis, which was lower than that observed for hand-sewn or circular stapled anastomosis. They described that the advantage of triangular stapled anastomosis was a lower frequency of anastomotic leakage and prevention of benign stenosis compared with other type of anastomotic techniques. In our case series, the anastomotic stenoses were observed in 12.5 % of patients, and the average number of endoscopic dilatations was 2.4. The triangulating stapled technique may be associated with a decreased frequency of postoperative anastomotic complications.

## Conclusions

In our modified triangulating stapling technique for esophagogastroanastomosis, all sides of anastomosis were performed in an everted fashion. The frequency of anastomotic leakage and benign stenosis were low. Our technique may present a feasible way to decrease anastomosis-related morbidity.
